# Supplementary material for: A Likelihood Approach for Real-Time Calibration of Stochastic Compartmental Epidemic Models
Source: PLoS Comput Biol. 2017 Jan 17;13(1):e1005257. doi: 10.1371/journal.pcbi.1005257 (PMC5240920; doi:10.1371/journal.pcbi.1005257)
Supplement: S1 File — (TAR.GZ) [file pcbi.1005257.s014.tar.gz › HSPH_Online-SI-Revision/MSS10/n10-extreme/n10-extreme_NEW2_summary2.pdf]

peak -8	3w spec	3w cum	5w spec	5w cum	end	3w spec LOG	3w cum LOG	5w spec LOG	5w cum LOG	end LOG
mean	147.7	144.	71.1	89.9	21.1	0.5	0.4	0.6	0.5	0.3
median	70.8	66.1	70.9	70.7	15.3	0.4	0.3	0.4	0.3	0.1
quantile .1	31.4	27.1	30.4	28.9	5.2	0.1	0.1	0.1	0.1	0.
quantile .9	190.7	171.5	103.4	118.7	43.4	1.2	0.9	1.5	0.1	0.8
33	33	27	28	34						

peak -4	3w spec	3w cum	5w spec	5w cum	end	3w spec LOG	3w cum LOG	5w spec LOG	5w cum LOG	end LOG
mean	18.3	16.4	21.3	15.8	20.2	0.1	0.1	0.1	0.1	0.1
median	14.6	14.3	12.7	12.2	10.	0.1	0.1	0.1	0.1	0.1
quantile .1	6.3	5.4	4.4	4.5	3.4	0.	0.	0.	0.	0.
quantile .9	32.8	29.2	55.7	34.1	49.8	0.2	0.2	0.4	0.	0.3
31	35	22	29	22						

peak	3w spec	3w cum	5w spec	5w cum	end	3w spec LOG	3w cum LOG	5w spec LOG	5w cum LOG	end LOG
mean	16.3	10.	24.7	12.8	17.8	0.1	0.	0.1	0.1	0.1
median	13.	7.8	17.9	9.5	13.6	0.1	0.	0.1	0.	0.1
quantile .1	4.5	1.9	8.8	2.4	3.8	0.	0.	0.	0.	0.
quantile .9	32.6	22.2	49.7	27.7	34.4	0.2	0.1	0.3	0.	0.2
16	14	16	8	5						

peak +4	3w spec	3w cum	5w spec	5w cum	end	3w spec LOG	3w cum LOG	5w spec LOG	5w cum LOG	end LOG
mean	21.4	17.1	29.	18.6	21.3	0.1	0.1	0.1	0.1	0.1
median	17.3	14.8	22.8	14.8	17.8	0.1	0.1	0.1	0.1	0.1
quantile .1	6.5	4.	10.	6.1	5.	0.	0.	0.	0.	0.
quantile .9	39.6	32.9	47.5	35.1	37.7	0.2	0.2	0.3	0.	0.2
12	8	21	7	6						

peak +8	3w spec	3w cum	5w spec	5w cum	end	3w spec LOG	3w cum LOG	5w spec LOG	5w cum LOG	end LOG
mean	55.8	46.6	65.6	48.4	52.7	0.3	0.2	0.3	0.2	0.2
median	30.9	36.6	41.4	35.2	34.8	0.1	0.1	0.2	0.1	0.1
quantile .1	10.5	6.7	15.3	7.4	6.4	0.	0.	0.1	0.	0.
quantile .9	134.9	97.3	110.	90.8	90.6	0.5	0.4	0.7	0.	0.4
17	3	20	4	2	{}	{}	{}	{}	{}	{}
